# Cost-Effectiveness of AI-Powered Decentralized Production for Advanced Therapies in the Hospital–Generated Chimeric Antigen Receptor T-Cell Therapy for Multiple Myeloma From a German Hospital Perspective: Early Health Technology Assessment Study

**DOI:** 10.2196/92253

**Published:** 2026-10-13

**Authors:** Iris W A Boot, Anna Louisa Weltin, Ysanne de Graaf, Hubertus J M Vrijhoef

**Affiliations:** 1 Panaxea 's-Hertogenbosch The Netherlands; 2 Department Bio-adaptive Production Fraunhofer Institute for Production Technology IPT Aachen, North Rhine-Westphalia Germany; 3 Faculty of Health, Medicine and Life Sciences Maastricht University Maatricht, Limburg The Netherlands

**Keywords:** cost-effectiveness analysis, chimeric antigen receptor T-cell therapy, CAR-T therapy, early health technology assessment, early HTA, artificial intelligence manufacturing, AI manufacturing, health economic modeling, automated decentralized manufacturing

## Abstract

**Background:**

Although effective, current chimeric antigen receptor T-cell (CAR-T) production methods—centralized, manual, and complex—are cost-intensive, time-consuming, and prone to variability. AIDPATH (AI-Powered Decentralized Production for Advanced Therapies in the Hospital) proposes a decentralized, automated alternative that integrates patient-specific data; optimizes resource use; and potentially improves cell viability, manufacturing efficiency, and patient outcomes.

**Objective:**

The aim of this study was to compare CAR-T therapy produced by the AIDPATH platform to both ciltacabtagene autoleucel (cilta-cel) and standard of care (SoC) for patients with triple-class refractory multiple myeloma (MM) over a 40-year time horizon from the hospital perspective in Germany.

**Methods:**

A partitioned survival model reflecting 3 health states (progression-free disease, progressed disease, and death) was used. The analysis used clinical trial data for cilta-cel, real-world data for SoC, and estimated parameters for AIDPATH due to the developmental status of the platform. The primary outcome was the incremental cost-effectiveness ratio; secondary outcomes included sensitivity and scenario analyses.

**Results:**

On the basis of the model used in this analysis, AIDPATH was dominant compared to both cilta-cel and SoC. Most costs for CAR-T therapies were driven by acquisition and adverse events. Sensitivity analyses showed that the results were most influenced by discount rates and assumptions about progression-free survival. Scenario analyses, including reduced adverse events and shorter vein-to-vein times for AIDPATH, further supported its cost-effectiveness.

**Conclusions:**

This is the first study to assess the cost-effectiveness of a CAR-T product generated with AI support in Germany from the hospital perspective. AIDPATH was found to be a cost-effective alternative to both cilta-cel and SoC, making it a promising option for future implementation. Although further data are needed, this study provides valuable guidance for health care stakeholders, reimbursement discussions, and future research.

## Introduction

Chimeric antigen receptor (CAR) T-cells are a new potent treatment option for patients with cancer, with long-term data demonstrating robust efficacy and overall low levels of toxicity. CAR T-cell (CAR-T) therapy works by collecting a patient’s T-cells, genetically modifying them to express tumor-targeting CARs, and reinfusing them to produce a targeted immune response [1]. However, as CAR-T therapy advances, a critical bottleneck is emerging in the manufacturing and distribution of these specialized cells [2].

Currently, CAR-T cells are predominantly manufactured in centralized facilities. This process introduces variability in the quality of the produced cells and limits scalability. Moreover, there is often a significant time gap between T-cell collection and CAR-T product infusion, referred to as the vein-to-vein time, which can lead to patient deterioration [2]. This can compromise product effectiveness or even render patients unsuitable for infusion. Although the standard production methods are groundbreaking, they are operationally complex and costly.

AIDPATH (AI-Powered Decentralized Production for Advanced Therapies in the Hospital) is a European Union–funded project (grant 101016909) addressing these challenges by automating and decentralizing CAR-T cell manufacturing. AIDPATH’s AI technology enables a deeper understanding of the production process and facilitates the identification of correlations between production and patient data, thereby supporting its patient-centric approach. The platform for producing this therapy is being built and is expected to optimize scheduling and resource planning, thereby reducing costs and hospital resource use. By overcoming the limitations of centralized manufacturing, AIDPATH has the potential to enhance efficiency, cost-effectiveness, and patient access to and outcomes of cancer treatment [3].

The objective of this study was to assess the cost-effectiveness of CAR-T cell therapy produced by the AIDPATH platform for patients with multiple myeloma (MM) in Germany, from the hospital perspective, over a 40-year time horizon, compared to manually produced and centrally distributed CAR-T cell therapy (ciltacabtagene autoleucel [cilta-cel]) and standard of care (SoC).

The results of this analysis will provide insights into whether transitioning to automated AI-supported CAR-T cell production is expected to be a cost-effective alternative. These results may support evidence-based decision-making for policymakers, health care administrators, and clinicians considering adoption of the AIDPATH platform.

## Methods

### Study Design and Population

To assess the cost-effectiveness of CAR-T cell therapy generated by the AIDPATH platform compared to SoC and manually produced, centrally distributed CAR-T cell therapy (cilta-cel; Johnson & Johnson), a partitioned survival model was developed as part of this early health technology assessment (HTA). The partitioned survival model consists of health states that a patient can occupy at a given time point, which reflect the clinical trajectory of patients [4].

The study population comprised adult patients with MM in Germany who had not responded to or had experienced a relapse/refractory (R/R) after at least 3 prior lines of cancer treatment. These treatments include an immunomodulator, a proteasome inhibitor, and a cluster of differentiation 38 antibody. In cases where the disease no longer responds to agents in each of the 3 classes, it is referred to as “triple-class refractory” MM [5]. This patient group is currently eligible for CAR-T cell therapy within the German health care system [6].

Given that social, environmental, and other contextual factors influence disease progression [7], this study focuses on a single country. As the first AIDPATH platform is being implemented at the University Hospital Würzburg, a leading center for CAR-T cell research and treatment, Germany was selected as the setting for this analysis.

### Model Overview

The partitioned survival model used in this study consists of 3 health states: progression-free disease (PFD), progressive disease (PD), and death (Figure 1). For the cohort receiving cilta-cel, the starting point was “apheresis” on the decision tree. Hereafter, patients were either not infused and streamed into the partitioned survival model or started a bridging period while they waited for their CAR-T therapy to be produced. After the bridging period, the infused cohort streamed into the partitioned survival model. All cohorts start in the PFD health state. Patients enter the model in the PFD health state and receive either CAR-T cell therapy (derived from AIDPATH or cilta-cel) or SoC. Patients enter the model at 61 years old, based on the selected patient group’s average age of 61.7 years in the cilta-cel clinical trial [8]. A cycle length of 1 week was used, and the time horizon for the analysis was 40 years (ie, 2080 cycles). This is in line with the Dutch Health Care Institute (*Zorginstituut Nederland* [*ZIN*]) cilta-cel study and represents a lifelong time horizon [5].

The number of patients in the PD health state at a certain time equals the overall survival (OS) proportion minus the progression-free survival (PFS) proportion. The number of patients in the death state equals 1–OS. OS and PFS rates for SoC were based on German real-world clinical population data. Here, time to next treatment was used as a proxy for OS [9]. The OS and PFS rates for the CAR-T therapy arms were based on Kaplan-Meier curves (median OS was not reached at the data cutoff; 27.7-month PFS rate: 54.9%) digitized from published clinical trial data [8-10] and reconstructed into pseudoindividual patient data to enable parametric survival modeling. Parametric survival models (including exponential, Weibull, Gompertz, gamma, log-logistic, log-normal, and generalized gamma distributions) were fitted to the reconstructed data to enable extrapolation beyond the observed trial period. Model selection was based on statistical goodness of fit (Akaike information criterion [AIC]), visual inspection of fitted vs observed Kaplan-Meier curves, and assessment of hazard functions. The analyses were then used to extrapolate the survival data beyond the time frame covered by clinical trials. Detailed methodology for extrapolation, data fitting, and model selection can be found in Section A in Multimedia Appendix 1 [8,11-41].

The mortality rate for the PFD health state equals background mortality, retrieved from German life tables, obtained from the World Health Organization [42]. The mortality rate for the PD health state is cancer-specific mortality. The life tables used were from the most recent year available (2019) and for ages 61 to 100 years.

**Figure 1:**
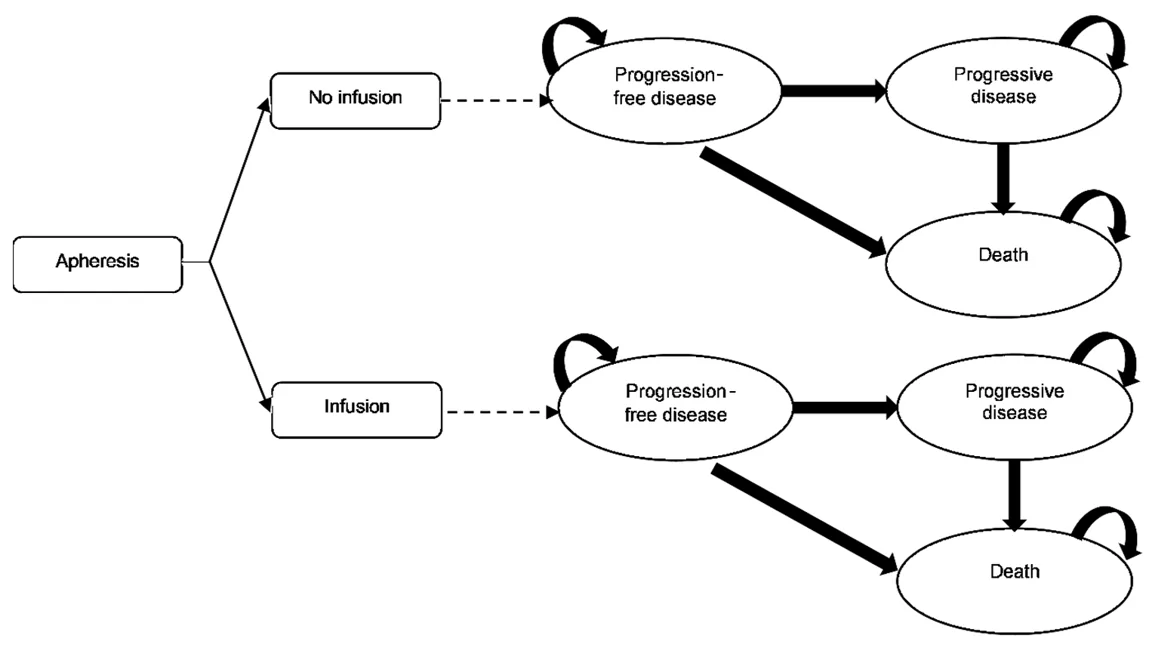
Partitioned survival model pathways and health states.

Data on health state utilities were derived from the CARTITUDE study [8] and the Dutch Health Care Institute (*ZIN*) cilta-cel study [5]. In these studies, the EQ-5D-5L questionnaire was used to assess utility scores, and mixed-effects models were used to estimate utilities for the PD state when data were missing [5]. Utilities were not age-adjusted. Data on efficacy and safety from the SoC therapies and cilta-cel CAR-T therapy were collected from publicly available trial results. Data from the AIDPATH platform will be collected once it is set up. When data are not available from the AIDPATH platform, estimates are based on cilta-cel.

Assuming similar clinical outcomes (PFS and OS), adverse event rates, and manufacturing success rates for AIDPATH and cilta-cel was an intentionally conservative modeling choice. In the base case, the manufacturing failure rate for AIDPATH was assumed to be equal to that of cilta-cel (3%) [43]. Manufacturing failures were not modeled as separate clinical events; instead, their impact was incorporated through increased production costs, reflecting the need for repeated manufacturing attempts to achieve a successful infusion. This assumption was made despite the AIDPATH platform incorporating a highly automated and standardized manufacturing process designed to reduce operator-dependent variability and production errors.

Consequently, assuming equivalent performance likely underestimates the potential clinical and operational advantages of the AIDPATH platform.

The costs for cilta-cel are based on the list price of €420,000 (€1=US $1.13; 2025 annual average) [5]. These costs are applied once. As AIDPATH does not have marketing authorization (yet), costs were based on production costs plus an estimation of marketing costs, totaling €120,000. The total cost of AIDPATH treatment was estimated at €120,000 based on a bottom-up aggregation of key cost components informed by published literature. Manufacturing (production) costs were assumed to be approximately €60,000 per treatment [11,44]. A 30% markup (€20,000) was applied to account for sales and marketing expenses [45]. Hospital-related costs were estimated at €27,000 [46], and quality control costs were approximately €13,000 [47]. Preinfusion period costs consisted of apheresis, bridging therapy, and conditioning therapy; these applied to both types of CAR-T therapies. Refer to Table S1 in Section B of Multimedia Appendix 1 for the cost breakdown.

Hospital resource use was based on data from the CARTITUDE-1 study [8], *ZIN* Carvykti economic analysis [5], and the AIDPATH consortium. Hospital costs and medication costs were based on the German fee tariff schedule (2023) [12], the *Kassenärtzliche Bundesvereinigung*, and publicly available economic evaluations [5,6] (Table 1). All costs were indexed to 2025 euros based on German inflation [48].

**Table 1:** Baseline parameters.

Parameters				Base case		Lower limit^a^		Upper limit		References
**Utilities**										
	Pre-infusion period			0.763		0.675		0.841		[5]
	PFS^b^ (on treatment)			0.804		0.721		0.875		[5]
	PFS (off treatment)			0.763		0.675		0.841		[5]
	Progressed disease			0.653		0.558		0.743		[5]
	Death			0		0		0		—^c^
	Disutility CAR-T^d,e^			1.286		—		—		—
	Disutility SoC^f^			0.225		0.149		0.311		—
**General information**										
	Time horizon (years)			40		10		50		[5]
	Discount rate outcomes (%)			3		0		5		[49]
	Discount rate costs (%)			3		0		5		[49]
	Average age (years)			61.70		37.51		85.89		[8]
	Average body weight (kg)			80.7		49.07		112.33		[8]
	Average body surface area (m^2^)			1.91		1.16		2.66		[8]
	AIDPATH^g^ vein-to-vein time (days)			26		16		36		—
	Cilta-cel vein-to-vein time (days)			51		31		71		[8]
**Costs (€, 2025)^h^**										
	Leukapheresis			2612.00		—		—		[11]
	**AIDPATH treatment**			120,000.00		—		—		—
		Bridging chemotherapy (12 days)	3058.64		—		—		[8,13]	
		Conditioning lymphodepleting chemotherapy acquisition	1178.22		—		—		[6]	
	**Cilta-cel treatment**			420,000.00		—		—		[5]
		Bridging chemotherapy (32 days)	—		—				[8,13]	
		Conditioning lymphodepleting chemotherapy acquisition	—		—				[6]	
	Administration lymphodepleting therapy			961.76		—		—		[14]
	NUB^i^-fee for CAR-T infusion			2035.00		—		—		—
	SoC drugs per week^j^			3506.10		—		—		—
	Fifth line treatment (one time)			9087.80		—		—		[9,50,51]
	Pre-infusion costs AIDPATH^k^			5198.62		—		—		—
	Pre-infusion costs Cilta cel^k^			10,243.94		—		—		—
	**Hospital stay**									
		Inpatient care per day	186.76		—		—		[12]	
		Intensive care unit	1831.53		—		—		[12]	
		day care unit hematology and internal oncology	406.98		—		—		[12]	
	Palliative care/end of life			8163.92		—		—		[52]

^a^Lower and upper limits represent the values used in sensitivity analyses to characterize uncertainty around the base-case parameter estimate.

^b^PFS: progression-free survival.

^c^Not applicable.

^d^CAR-T: chimeric antigen receptor T-cell.

^e^Total average of treatment, spread over multiple cycles.

^f^SoC: standard of care.

^g^AIDPATH: AI-Powered Decentralized Production for Advanced Therapies in the Hospital.

^h^€1=US $1.13 (2025 annual average).

^i^NUB: Neue Untersuchungs- und Behandlungsmethoden (new diagnostic and treatment methods).

^j^Detailed calculation is provided in Section B in Multimedia Appendix 1.

^k^Detailed calculation is provided in Section C in Multimedia Appendix 1.

SoC encompasses the physician’s choice of treatment for patients with triple-class refractory MM. These data were obtained from the German Therapy Monitor, a registry of real-world oncology data. It contains anonymized data on patients with approximately 4000 R/R MM, obtained from a representative sample of 108 German centers. Refer to Table S2 in Section C in Multimedia Appendix 1 for an overview of included drugs and Table S3 in Section C in Multimedia Appendix 1 for detailed calculations.

When patients enter the PD health state, costs for follow-up treatment were applied once for all treatments. The treatment given as fifth-line R/R MM treatment is one of the regimens given in the fourth line that the patient has not received before [9]. Therefore, the average fifth-line treatment price for SoC and CAR-T is the same as that for the fourth line. The average treatment duration in the fifth line is assumed to be 4 months [50]. Monthly costs of fifth-line treatment are €10,349.98. The percentage of patients with fourth-line R/R MM that receive fifth-line treatment is 0.22, according to attrition rates in a study by Dhanasiri [51]. This results in the applied 1-time follow-up treatment costs being €9108.00 (4×€10,349.98×0.22).

All baseline parameters are presented in Table 1.

The primary outcome of the analyses is the incremental cost-effectiveness ratio (ICER), which is calculated by dividing the difference in total costs (incremental cost) by the difference in quality-adjusted life years (QALYs; incremental effect) [53].

Secondary outcomes include the avoidance of adverse events. Adverse events were only included if they incurred health care costs (required physician input and/or hospitalization and/or medication) or had a large impact on patient quality of life (refer to Table S4 in Section D in Multimedia Appendix 1 for a list of included adverse events). As the occurrence rates of adverse events for AIDPATH are not yet available, they are assumed to be equal to those of cilta-cel.

Following the methodological guidelines of the German National Institute for Quality and Efficiency in Health Care (*Institut für Qualität und Wirtschaftlichkeit im Gesundheitswesen* [*IQWiG*]), the German institute responsible for evaluating health care interventions, outcomes, and costs was discounted at 3% [49].

The model was constructed using Excel (version 2608; Microsoft Corp).

### Sensitivity Analyses

A 1-way sensitivity analysis was performed to assess the model results for robustness and to identify the main cost drivers. Each parameter was varied by 20% [54]. Refer to Table 1 for the lower- and upper-limit values.

### Scenario Analyses

Various scenarios were performed to assess the economic implications of the competing treatment strategies and to test the robustness of the different assumptions made in the model.

In the first scenario analysis, the risk of adverse events following AIDPATH-produced CAR-T cell therapy was decreased by 10% and 20%. CAR-T–specific side effects after AIDPATH-produced CAR-T cell infusion are expected to decrease. Furthermore, the CARs are equipped with an epidermal growth factor receptor marker, which may prevent fatal outcomes of CAR-T cell–related toxicity [55].

In the second scenario analysis, the shipping and manufacturing time of CAR-T cells produced by the AIDPATH platform was lowered. In this scenario, we assumed the manufacturing time (14 days) plus shipping time (0 days) as the bridging period for AIDPATH (the average bridging therapy duration for cilta-cel is 32 days). A shorter bridging period is expected to reduce the incidence of bridging-related adverse events. It also affects the number of patients who remain alive and eligible to receive lymphodepleting therapy and the subsequent CAR-T infusion. Because bridging therapy is shorter, the direct costs of bridging treatment decrease, along with the costs of managing adverse events associated with bridging chemotherapy.

To explore uncertainty in important assumptions related to AIDPATH, additional scenario analyses were performed. In the third scenario, the PFS rate of AIDPATH was varied by ±10% to assess the impact of potential differences in treatment efficacy. In the fourth scenario, the production costs of AIDPATH were increased by 10% and 20% to reflect a higher manufacturing failure rate. An increase in manufacturing failure rate implies that more production attempts are required per successfully treated patient, thereby increasing the cost per treatment. This approach allows an indirect assessment of the impact of manufacturing failure on cost-effectiveness outcomes.

To address uncertainty in the AIDPATH cost estimate, a fifth scenario analysis was added to explore the impact of the total costs of an AIDPATH treatment. To do so, costs were varied to €200,000 and €300,000.

### Ethical Considerations

This study presents an early HTA based exclusively on secondary data obtained from previously published literature, publicly available sources, and aggregated data. No primary data were collected, and no individual-level patient data were accessed.

According to German and Dutch regulations governing research involving human participants, ethics approval from an ethics committee (*Ethikkommission*) is not required for studies that rely solely on anonymized, aggregated, or publicly available data and do not involve interventions with human participants [56,57]. Therefore, formal ethics approval and informed consent were not required for this study.

## Results

The mixture-cure log-normal distribution was selected for the extrapolation of PFS (AIC=324.56) and OS (AIC=431.23) data (refer to Figures S1 and S2 and Tables S1-S3 in Section E in Multimedia Appendix 1 for the results of the selection process).

### Primary Result

The results indicate that AIDPATH-produced CAR-T cell therapy is projected to be both more effective and less costly than current SoC and cilta-cel treatment. Specifically, patients receiving AIDPATH therapy are expected to gain more QALYs while incurring lower overall treatment costs. This improvement in cost-effectiveness is reflected in the ICERs, which are negative for both comparisons: –€3075.08 when comparing AIDPATH to SoC and –€1,986,526.56 when comparing AIDPATH to cilta-cel. A negative ICER indicates that AIDPATH therapy dominates the alternatives, meaning it provides better health outcomes at a lower cost. Detailed results are presented in Table 2.

Most costs of the CAR-T cell therapies can be attributed to the acquisition, followed by adverse events and monitoring (Table 3).

**Table 2:** Primary outcomes in the base case analysis.

Treatments	Costs (€)^a^	Quality-adjusted life years	Incremental cost-effectiveness ratio (AIDPATH^b^ compared to alternative)	Adverse event costs (€)^a^
AIDPATH	167,895.77	5.10	Reference	24,131.66
Cilta-cel	436,967.32	4.96	–1,986,526.56	24,131.66
Standard of care	180,114.69	1.13	–3075.08	4722.48

^a^€1=US $1.13 (2025 annual average).

^b^AIDPATH: AI-Powered Decentralized Production for Advanced Therapies in the Hospital.

**Table 3:** Cost breakdown of primary results.

Treatments	Acquisition of treatment (€)^a^	Administration of treatment (€)^a^	Monitoring of patient (€)^a^	Adverse event (€)^a^	Follow-up treatment (€)^a^	End of life (€)^a^
AIDPATH^b^	113,216.30	1927.01	12,864.01	24,131.66	9087.80	6668.99
Cilta-cel	382,779.98	1863.47	12,388.68	24,131.66	9087.80	6715.74
Standard of care	150,019.83	6125.16	2214.26	4772.48	9087.80	7898.01

^a^€1=US $1.13 (2025 annual average).

^b^AIDPATH: AI-Powered Decentralized Production for Advanced Therapies in the Hospital.

### Sensitivity Analyses

An overview of the model input parameters with the largest impact on the ICER when comparing AIDPATH-produced CAR-T cell therapy to SoC is shown in Figure S3 and Table S8 in Section F in Multimedia Appendix 1. The results show that the discount rate of outcomes and PFS rate have the largest impact on the ICER. This indicates that assumptions about the valuation of future health benefits and long-term patient outcomes play a critical role in determining the overall cost-effectiveness of AIDPATH therapy.

An overview of the model input parameters with the largest impact on the ICER, when comparing AIDPATH therapy to cilta-cel CAR-T cell therapy, is shown in Figure S4 and Table S9 in Section F in Multimedia Appendix 1. The results show that the discount rate of outcomes and the vein-to-vein time have the largest impact on the ICER. This suggests that both the assumptions regarding how future health benefits are valued over time and the time required for manufacturing and delivering the CAR-T cell product play a crucial role in determining the relative cost-effectiveness of AIDPATH therapy compared to cilta-cel.

### Scenario Analyses

The scenario analyses demonstrated that the results were generally robust to variations in key assumptions. Reducing adverse event risks (scenario 1) slightly lowered costs, with QALYs unchanged, and AIDPATH remained dominant vs both comparators. Shortening manufacturing and shipping times (scenario 2) increased QALYs with modest cost increases; AIDPATH remained dominant compared to cilta-cel but was less cost-saving compared to SoC.

Varying PFS rates (scenario 3) affected both costs and QALYs; however, AIDPATH remained dominant across all variations. Increasing production costs to reflect lower manufacturing success rates (scenario 4) raised total costs without affecting health outcomes. Despite these increases, AIDPATH remained cost-effective compared with cilta-cel and SoC. Increasing treatment prices (scenario 5) substantially increased costs (Table 4).

**Table 4:** Results of scenario analyses.

Scenarios and treatments				Costs (€)^a^		Quality-adjusted life years		Incremental cost-effectiveness ratio (compared to SoC^b^)	AE^c^ costs (€)
**Scenario 1: lower risk of AEs with AIDPATH^d^ CAR-T^e^ therapy**									
	**AIDPATH** –**10%**		165,518.17		5.10		Reference		21,754.06
		Cilta-cel	436,967.32		4.96		–1,938,922.50		24,131.66
		SoC	180,114.69		1.13		–3676.71		4722.48
	**AIDPATH** –**20%**		163,132.67		5.10		Reference		19,368.56
		Cilta-cel	436,967.32		4.96		–1,955,961.79		24,131.66
		SoC	180,114.69		1.13		–4277.59		4722.48
**Scenario 2: shorter shipping and manufacturing time of AIDPATH CAR-T**									
	AIDPATH		172,296.18		5.25		Reference		24,131.66
	Cilta-cel		436,967.32		4.96		–912,659.10		24,131.66
	SoC		180,114.69		1.13		1897.70		4722.48
**Scenario 3: variation in progression-free survival rates for AIDPATH treatment**									
	**AIDPATH –10%**		154,277.14		5.01		Reference		24,131.66
		Cilta-cel	436,967.32		4.96		–5,653,803.60		24,131.66
		SoC	180,114.69		1.13		–6659.16		4722.48
	**AIDPATH +10%**		168,610.22		5.15		Reference		24,131.66
		Cilta-cel	436,967.32		4.96		–1,412,405.79		24,131.66
		SoC	180,114.69		1.13		–2861.81		4722.48
**Scenario 4: variation in manufacturing success rate (ie, variation in production costs)**									
	**AIDPATH +10%**		173,556.58		5.10		Reference		24,131.66
		Cilta-cel	436,967.32		4.96		–1,881,505.29		24,131.66
		SoC	180,114.69		1.13		–1651.92		4722.48
	**AIDPATH +20%**		179,217.40		5.10		Reference		24,131.66
		Cilta-cel	436,967.32		4.96		–1,841,070.86		24,131.66
		SoC	180,114.69		1.13		–226.02		4722.48
**Scenario 5: increased costs of AIDPATH CAR-T treatment**									
	**AIDPATH (€200,000)**		243,373.30		5.10		Reference		24,131.66
		Cilta-cel	436,967.32		4.96		–1,382.814.43		24,131.66
		SoC	180,114.69		1.13		15,934.16		4722.48
	**AIDPATH (€300,000)**		337,720.22		5.10		Reference		24,131.66
		Cilta-cel	436,967.32		4.96		–708,907.86		24,131.66
		SoC	180,114.69		1.13		36,699.13		4722.48

^a^€1=US $1.13 (2025 annual average).

^b^SoC: standard of care.

^c^AE: adverse event.

^d^AIDPATH: AI-Powered Decentralized Production for Advanced Therapies in the Hospital.

^e^CAR-T: chimeric antigen receptor T-cell.

## Discussion

### Principal Findings

The objective of this study was to assess the cost-effectiveness of CAR-T cell therapy produced by the AIDPATH platform for patients with triple-class refractory MM in Germany, compared to manually produced and centrally distributed CAR-T cell therapy (cilta-cel) and SoC. In this model, given its assumptions and from a hospital perspective, AIDPATH CAR-T therapy was shown to be dominant over a period of 40 years, offering improved outcomes and lower overall costs than both comparators. Despite the inferiority, only a small difference was observed in QALY outcomes between the CAR-T treatments in the base case analysis. This is despite the expectation that shorter vein-to-vein time would increase the proportion of patients remaining eligible for infusion by limiting progression during the manufacturing period [2]. Due to limited evidence, equal eligibility was conservatively assumed, and thus, the observed QALY difference primarily reflects earlier treatment administration rather than differences in infusion eligibility.

The findings suggest that the AIDPATH platform could be considered for implementation within the German health care setting. The results may provide a basis for estimating the return on investment for relevant stakeholders. Importantly, the base case assumptions were intentionally conservative. Equivalent clinical outcomes (PFS and OS), adverse event rates, and manufacturing failure rates were assumed for AIDPATH and cilta-cel, despite the expectation that AIDPATH’s automated and standardized manufacturing process may reduce variability and production errors in practice. The scenario analyses, which included further conservative adjustments, such as increased costs and limited efficacy gains, demonstrated that AIDPATH remained cost-effective, supporting the robustness of the findings.

The sensitivity analyses illustrate the sensitivity of the results to changes in methodological choices. In both sensitivity analyses, only a few parameters had a large impact on the outcomes. These parameters are important to consider when modifying or scaling up the platform to safeguard its cost-effectiveness compared to alternative treatment strategies.

The results of this study can be used to guide discussions with German stakeholders, including investors and policymakers, regarding the incremental cost-effectiveness of CAR-T cell therapy and reimbursement decisions. Furthermore, the findings can be considered exemplary for countries with comparable health care systems. Finally, AIDPATH uses a patient-centric approach to CAR-T cell production, and based on the results, this approach shows potential.

### Strengths and Limitations

To our knowledge, this is the first early HTA study to assess the cost-effectiveness of AI-supported CAR-T cell therapy production in Germany from the hospital perspective. This study is tailored to the German health care setting and provides a model that can be updated when new data become available.

Although this cost-effectiveness study provides valuable insights to support decision-making, it has several important limitations that must be acknowledged. First, by focusing narrowly on cost-effectiveness, the study may overlook the broader potential benefits of automated CAR-T therapy production. These may include system-level advantages associated with decentralized manufacturing, such as reduced logistical complexity, lower risks related to cold chain management and cross-border shipment, and improved access to treatment through reduced patient travel or expanded treatment capacity. In addition, automated production may improve treatment convenience and patient satisfaction and streamline clinical workflows. These qualitative outcomes can significantly impact health care delivery and patient experience but are often difficult to adequately quantify in economic evaluations [58]. Moreover, as with many applications of medical AI, there are pressing concerns about appropriate regulation and evaluation [59]. HTA has been recognized as a potentially key method for evaluating AI-driven innovations. However, this model does not fully address AI-specific challenges, such as the assessment of continuously learning systems, the explainability of AI decision-making, and interoperability with existing health care infrastructure. To address these gaps, Boverhof et al [59] proposed a comprehensive framework of 29 evaluation elements across 4 domains, developed through a systematic and targeted review, which could serve as a more nuanced approach for assessing the value of AI in medicine. This broader perspective is crucial for ensuring that automated CAR-T cell production and similar technologies are appraised not only for their economic impact but also for their systemic and societal implications.

Second, no specific data regarding the safety and effectiveness of CAR-T therapy generated by the AIDPATH platform are available yet. The platform is currently being tested, and it is important to update the model as soon as clinical trial data become available. In addition, survival extrapolation is an important source of uncertainty in this analysis. A mixture-cure log-normal model was selected based on statistical goodness-of-fit, visual inspection, and clinical plausibility. Alternative parametric models were explored during model selection; however, these showed inferior fit and/or less plausible long-term extrapolations.

Third, a limitation in achieving the objective of this study is the reliance on Dutch CARTITUDE trial data for key comparator inputs, specifically for the centrally manufactured CAR-T therapy cilta-cel [5]. Due to the lack of German-specific data, key cost and resource use parameters for cilta-cel were derived from a Dutch study, and these values were also used as a proxy for the AIDPATH-generated CAR-T cell therapy. However, this approach introduces uncertainty, as health care delivery, cost structures, and reimbursement mechanisms differ between the Dutch and German hospital systems. For example, hospital tariffs, staff costs, and care pathways in Germany may be higher or more complex, potentially leading to an underrepresentation of true costs from the German hospital perspective. This could result in a more favorable cost-effectiveness profile for CAR-T cell therapies than would be observed with German-specific data. Future studies should aim to validate these inputs with empirical data from the German context to strengthen the reliability of the cost-effectiveness conclusions. Because this approach was used for both CAR-T cell therapy arms, it is unlikely that an updated model would change the conclusions of the current study.

Despite these limitations, an HTA at this early stage is considered highly valuable because it provides critical insights into the potential clinical and economic impact of this new medical product. It supports informed decision-making during early development stages, helps prioritize investments, guides research efforts, and identifies key data gaps to address before implementation [60].

### Conclusions

This study is the first to assess the cost-effectiveness of AI-supported, hospital-based CAR-T cell therapy produced by the AIDPATH platform for patients with MM in Germany from the hospital perspective. On the basis of the assumptions inherent in this model, AIDPATH therapy was found to be cost-effective and dominant compared with both cilta-cel and SoC from the hospital perspective over a 40-year time horizon. This was found in the base case analysis and was confirmed by all scenario analyses performed.

However, key inputs were partly based on Dutch data due to the lack of German-specific figures, introducing uncertainty, particularly in cost estimates. Additionally, no clinical data are yet available for AIDPATH-generated CAR-T cell therapy, and broader benefits, such as patient satisfaction and system efficiency, were not captured in the cost-effectiveness analysis.

Despite these challenges, this early HTA provides valuable insights to guide further research, inform reimbursement discussions, and support planning for future implementation in Germany and similar health care systems.

## Appendix

Multimedia Appendix 1 A: Methodology for extrapolation, data fitting, and model selection of overall survival (OS) and progression-free survival (PFS) data; B: preinfusion costs; C: standard of care drugs; D: adverse events; E: results of extrapolation, data fitting, and model selection for OS and PFS data; and F: scenario analysis results.

## Abbreviations

AIC: Akaike information criterion
AIDPATH: AI-Powered Decentralized Production for Advanced Therapies in the Hospital
CAR: chimeric antigen receptor
CAR-T: chimeric antigen receptor T-cell
Cilta-cel: ciltacabtagene autoleucel
HTA: health technology assessment
ICER: incremental cost-effectiveness ratio
IQWiG: Institut für Qualität und Wirtschaftlichkeit im Gesundheitswesen
MM: multiple myeloma
OS: overall survival
PD: progressive disease
PFD: progression-free disease
PFS: progression-free survival
QALY: quality-adjusted life year
R/R: relapse/refractory
SoC: standard of care
ZIN: Zorginstituut Nederland

## Data Availability

All data generated or analyzed during this study are included in this published article and its supplementary information files.
