# Supplementary material for: Cost-Effectiveness of AI-Powered Decentralized Production for Advanced Therapies in the Hospital–Generated Chimeric Antigen Receptor T-Cell Therapy for Multiple Myeloma From a German Hospital Perspective: Early Health Technology Assessment Study
Source: JMIR Cancer. 2026 Oct 13;12:e92253. doi: 10.2196/92253 (PMC13718277; doi:10.2196/92253)
Supplement: Supplementary file 1 [file cancer_v12i1e92253_app1.docx]

Table of contents

[Appendix A: Methodology for extrapolation, data fitting, and model selection of overall survival (OS) and progression free survival (PFS) data Overall survival and progression free survival data	1](#_Toc240895450)

[Appendix B: Pre-infusion costs	2](#_Toc240895451)

[Appendix C: Standard of care drugs	4](#_Toc240895452)

[Appendix D: Adverse events	8](#_Toc240895453)

Appendix E: Results of extrapolation, data fitting, and model selection for OS and PFS
 data ………………………………………………………………………………………………….11

Appendix F: Scenario analysis results …………………………………………………………...13

# Appendix A: Methodology for extrapolation, data fitting, and model selection of overall survival (OS) and progression free survival (PFS) data Overall survival and progression free survival data

**Digitization of survival data and model selection**

Kaplan–Meier curves from clinical research were digitized to obtain pseudo-individual patient data (IPD) in R (version 4.3.0). The accuracy of digitization was validated by comparing median overall survival (OS) between the clinical trials and the generated pseudo-IPD and with visual inspection between the generated and published Kaplan–Meier curves. The choice of parametric models for extrapolation in the survival analysis was based on established guidelines recommended by the National Institute for Health and Care Excellence Decision Support Unit Technical Support Documents 14 and 21 (1, 2). The models considered include the Exponential, Weibull, Gompertz, Gamma, Log-logistic, Lognormal, and Generalized Gamma distributions. The best-fitting parametric model was selected by:

- Inspection of log cumulative hazard plots and their fit to the smoothed hazard,^,^
- Visual fit of the survival curves, and
- Statistical comparison using the Akaike Information Criterion (AIC) (3).

**Extrapolation of survival data**

OS and Progression-Free Survival (PFS) are measures commonly used in economic analyses. OS measures time from treatment initiation to death, while PFS tracks time until disease progression or death. These metrics are derived from cancer trials and provide critical insights into the treatment's impact on patient outcomes. To capture the full benefit of therapies, long-term extrapolation is necessary, as requested by health technology assessment (HTA) agencies like NICE.

Survival in each cycle of the model is calculated as:

$$proportion alive according to chosen model in previous cycle*proportion alive according to chosen model in current cycle$$

Survival is set to zero if the population age exceeds 100 (age cutoff). If background mortality exceeds OS, the model switches to background mortality.

1

4

# Appendix B: Pre-infusion costs

**Table S4**: Detailed calculation pre-infusion costs AIDPATH and Cilta cel

2

| Cost category | Unit | Number of units/ days | Proportion receiving | Cost per unit / per day (€, 2025) | Total Cost (€) | Cost per patient Cilta (€) | Cost per patient AIDPATH (€) | *Source* |
| --- | --- | --- | --- | --- | --- | --- | --- | --- |
| Leukapheresis procedure* | 1 | 0 | 1.00 | 2,612.00 | 0 | 0.00 | 0.00 | (4) |
| Cryopreservation (without storage and thawing)** | 1 | 0 | 0.79 | 534.00 | 0 | 0.00 | 0.00 | AIDPATH consortium |
| Bridging chemotherapy (Cilta-cel) | mg/kg per day | 32 | 0.77 | 331.00 | 10,593.69 | 8,156.37 |  | (5, 6) |
| Bridging chemotherapy (AIDPATH) | mg/kg per day | *12* | 0.77 | 331.00 | 3,972.26 |  | 3,058.64 |  |
| Conditioning lymphodepleting chemotherapy acquisition (Cilta-cel) | mg/m^2^ per day | 3 | 0.89 | 419.80 | 1,259.30 | 1,125.81 |  |  |
| Conditioning lymphodepleting chemotherapy acquisition (AIDPATH) | mg/m^2^ per day | 3 | 0.96 | 409.00 | 1,227.09 |  | 1,178.22 | (6) |
| Administration lymphodepleting therapy (both) |  |  |  |  | 961.76 | 961.76 | 961.76 |  |
| - *Outpatient* | *day* | *3* | *0.50* | *164.32* |  |  |  | (7) |
| - *Inpatient* | *Day* | *7* | *0.50* | *204.36* |  |  |  | (7) |
| *Total (€)* |  |  |  |  |  | ***10,243.94*** | ***5,198.62*** |  |

Abbreviations: AIDATH:

*: Leukapheresis is part of the production of the drug according to Section 4 Paragraph 14 AMG, there are no additional costs for the drug being evaluated: Manufacturing is the producing, preparing, formulating, treating or processing, filling as well as decanting, packaging, labelling and release of medicinal products (8)

**: Manufacturing is the producing, preparing, formulating, treating or processing, filling as well as decanting, packaging, labelling and release of medicinal products (8)

3

# Appendix C: Standard of care drugs

**Table S5:** Costs of SoC drugs

| Drug | Price  (€, 2025) | Amount | Unit | Administration | Source(s) |
| --- | --- | --- | --- | --- | --- |
| Bendamustine | 104.39 | 25 | mg | ampul | (9) |
| Bortezomib | 1,892.40 | 3,5 | mg | ampul | (10) |
| Carfilzomib | 760.88 | 30 | mg | powder for solution | (10) |
| Daratumumab | 6,498.11 | 1399 | mg | Gabe von Daratumumab, intravenös | (10) |
| Dexamethasone | 3.19 | 20 | mg | Tablet | (11) |
| Elotuzumab | 3,871.33 | 999 | mg | Gabe, parental | (10) |
| Ixazomib | 2,152.31 | 4 | mg | Capsule, same for 3/2.3 mg | GFT |
| Lenalidomide | 452.16 | 25 | mg | Capsule | (10) |
| Melphalan | 4.06 | 2 | mg | Tablet | (12) |
| Panobinostat | 776.48 | 20 | mg | Capsule, same for 10/15/20 mg | GFT |
| Pomalidomide | 436.64 | 4 | mg | Capsule | GFT |
| Prednisone | 0.36 | 20 | mg | Tablet | (12) |
| Thalidomide | 21.78 | 50 | mg | Capsule | (11) |

4

**Table S6**: SoC drug regimens, costs per week calculation

5

| Regimen name | Drugs | Mg needed per dosage*, including waste | Costs per dosage (€, 2025) | Surcharge preparation (€) | Number of administrations in cycle | Costs per cycle (€, 2025) | Cycle length (days) | Cost per week (€, 2025) | Percentage in RWCP (%) | Source(s) |
| --- | --- | --- | --- | --- | --- | --- | --- | --- | --- | --- |
| MelPred | Melphalan | 18.00 | 36.55 | 0.00 | 4 | 146.18 | 35 | **29.23** | 11.373 | (13) |
|  | Prednisone | 115.00 | 2.14 | 0.00 | 4 | 8.56 | 35 | **1.71** |  |  |
| VRd (BorLenDex) | Bortezomib | 3.50 | 1,892.40 | 81.00 | 4 | 7,893.58 | 21 | **2,631.19** | 0 | (14) |
|  | Lenalidomide | 25.00 | 452.16 | 0.00 | 14 | 6,330.17 | 21 | **2,110.06** |  |  |
|  | Dexamethasone | 20.00 | 3.19 | 0.00 | 8 | 25.56 | 21 | **8.52** |  |  |
| Dar | Daratumumab |  |  |  |  |  |  |  |  | (15) |
|  | Cycle 1-2 | 1292 | 6,498.11 | 71.00 | 4 | 26,276.45 | 28 | **6,569.11** | 7.618 |  |
|  | Cycle 3-6 | 1292 | 6,498.11 | 71.00 | 2 | 13,138.22 | 28 | **3,284.56** | 7.618 |  |
|  | Cycle 7-13 | 1292 | 6,498.11 | 71.00 | 1 | 6,569.11 | 28 | **1,642.28** | 7.618 |  |
| ThalPred | Thalidomie | 50 | 21.78 | 81.00 | 28 | 2,877.72 | 28 | **719.43** | 4.506 | (16) |
|  | Prednisone | 77 | 1.43 | 0 | 7 | 9.99 | 28 | **2.50** |  |  |
| IxaRd | Ixazomib | 4 | 2,152.31 | 0 | 3 | 6,456.94 | 28 | **1,614.24** | 18.884 | (17) |
|  | Lenalidomide | 25 | 452.16 | 0 | 21 | 9,495.26 | 28 | **2,373.81** |  |  |
|  | Dexamethasone | 40 | 6.39 | 0 | 4 | 25.56 | 28 | **6.39** |  |  |
| LenDex | Lenalidomide | 25 | 452.16 | 0 | 21 | 9,495.26 | 28 | **2,373.81** | 2.361 | (18) |
|  | Dexamethasone | 40 | 6.39 | 0 | 4 | 25.56 | 28 | **6.39** |  |  |
| Vd | Bortezomib |  |  |  |  |  |  |  |  | (13) |
|  | Cycle 1-2 | 3.5 | 1,892.40 | 81.00 | 4 | 7,893.58 | 21 | **2,631.19** | 7.618 |  |
|  | Cycle 3-13 | 3.5 | 1,892.40 | 81.00 | 4 | 7,893.58 | 21 | **1,578.72** | 7.618 |  |
|  | Dexamethasone |  |  |  |  |  |  |  |  | (19) |
|  | Cycle 1-2 | 20 | 3,19 | 0 | 8 | 25.56 | 35 | **8.52** |  |  |
|  | Cycle 3-13 | 20 | 3.19 | 0 | 8 | 25.56 | 35 | **5.11** |  |  |
| Kd | Carfilzomib | 107 | 3,043.52 | 81.00 | 6 | 18,747.12 | 28 | **4,686.78** | 4.506 | (20) |
|  | Dexamethasone | 20 | 3.19 | 0 | 8 | 25.56 | 28 | **6.39** |  |  |
| KRd | Carfilzomib | 25 | 760.88 | 81 | 6 | 5,051.28 | 28 | **1,262.82** | 2.039 | (21) |
|  | Lenalidomide | 25 | 452.16 | 0 | 21 | 9,495.26 | 28 | **2,373.81** |  |  |
|  | Dexamethasone | 40 | 6.39 | 0 | 4 | 25.56 | 28 | **6.39** |  |  |
| EloRd | Elotuzumab |  |  |  |  |  |  |  |  | (22) |
|  | Cycle 1-2 | 807 | 3,871.33 | 71.00 | 4 | 15,769.33 | 28 | **3,9442.33** | 7.94 |  |
|  | Cycle 3-13 | 807 | 3,871.33 | 71.00 | 2 | 7,884.66 | 28 | **1,971.17** | 7.94 |  |
|  | Lenalidomide |  |  |  |  |  |  |  |  |  |
|  | Cycle 1-2 | 25 | 452.16 | 0 | 21 | 9,495.26 | 28 | **2,373.81** |  |  |
|  | Cycle 3-13 | 25 | 452.16 | 0 | 21 | 9,495.26 | 28 | **2,373.81** |  |  |
|  | Dexamethasone |  |  |  |  |  |  |  |  |  |
|  | Cycle 1-2 | 28 | 6.39 | 0 | 4 | 25.56 | 28 | **6.39** |  |  |
|  | Cycle 3-13 | 4 | 6.39 | 0 | 2 | 12.78 | 28 | **3.19** |  |  |
| PanoV | Panobinostat | 20 | 776.48 | 0 | 6 | 4,658.88 | 21 | **1,552.96** | 4.506 | (23)  6 |
|  | Bortezomib | 3.5 | 1,892.40 | 81.00 | 4 | 7,893.58 | 21 | **2,631.19** |  |  |
|  | Dexamethasone | 20 | 3.19 | 0 | 8 | 25.56 | 21 | **8.52** |  |  |
| PVd | Pomadolidomide | 4 | 776.48 | 0 | 14 | 10,870.73 | 21 | **3,623.57** | 1.395 | (24) |
|  | Bortezomib | 3,.5 | 1,892.40 | 81.00 | 4 | 7,893.58 | 21 | **2,631.19** |  |  |
|  | Dexamethasone | 20 | 3.19 | 0 | 8 | 25.56 | 21 | **8.52** |  |  |
| PomDex | Pomadolidomide | 4 | 776.48 | 0 | 21 | 16,306.09 | 28 | **4,076.52** | 16.202 | (23) |
|  | Dexamethasone | 40 | 6.39 | 0 | 4 | 25.56 | 28 | **6.39** |  |  |
| EloPd | Elotuzumab | 807 | 3,871.33 | 71.00 | 4 | 15,769.33 | 28 | **3,942.33** | 2.790 | (25) |
|  | Pomadolidomide | 4 | 776.48 | 0 | 21 | 16,306.09 | 28 | **4,076.52** |  |  |
|  | Dexamethasone | 40 | 6.39 | 0 | 4 | 25.56 | 28 | **6.39** |  |  |
| DVd | Daratumumab | 1292 | 6,498.11 | 71.00 | 3 | 19,707.34 | 21 | **6,569.11** | 3.755 | (13) |
|  | Bortezomib | 3,5 | 1,892.40 | 81.00 | 4 | 7,893.58 | 21 | **2,631.19** |  |  |
|  | Dexamethasone | 20 | 3.19 | 0 | 8 | 25.56 | 21 | **8.52** |  |  |
| DRd | Daratumumab | 1292 | 6,498.11 | 71.00 | 4 | 26,276.45 | 28 | **6,569.11** | 1.717 | (22) |
|  | Lenalidomide | 25 | 452.16 | 0 | 21 | 9,495.26 | 28 | **2,373.81** |  |  |
|  | Dexamethasone | 40 | 6.39 | 0 | 4 | 25.56 | 28 | **6.39** |  |  |
| BendaPred | Bendamustine | 230 | 1,043.85 | 0 | 2 | 2,087.71 | 28 | **521.93** | 2.790 | (26) |
|  | Prednisone | 115 | 2.14 | 0 | 28 | 59.94 | 28 | **14.98** |  |  |
|  | | | | ***Average costs per week of regimens***** | | | | ***€3,506.10*** |  | |

*: based on average m^2^

**: taking into account the different cycle costs and percentages of administration

7

Abbreviations: Mg: milligrams, RWCP: real-world clinical practice.

111=

# Appendix D: Adverse events

**Table S7:** Overview of adverse events

8

| Adverse event | Disutility | Probability CAR-T | Probability SoC | Costs per AE (€)^1^ (2025) | Duration (cycles) | Source |
| --- | --- | --- | --- | --- | --- | --- |
| Anaemia | 0.31 | 0.68 | 0.116 | 3,111.30 | 1.237 | (27) |
| ST increased | 0.07 | 0.052 |  | 406.72 | 0.514 | (28) |
| Asthenia and fatigue | 0.12 | 0.052 | 0.046 | 2,637.66 | 1.286 | (27) |
| Cytokine release syndrome grade 1/2 | 0.06 | 0.897 |  | 7,748.90 | 0.657 | Assumed to be the cost of tocilizumab, 8mg/kg for 4 doses, only for the 36% of this cohort with Grade 2 CRS *4 doses only in severe cases, but conservative estimation |
| Cytokine release syndrome grade 3/4 | 0.23 | 0.051 |  | 16,131.90 | 3.543 | Assumed to be the cost of tocilizumab, 8mg/kg for 4 doses, plus 4.5 days ICU |
| Neurological events grade 1/2 | 0.04 | 0.120 |  | 0.00 | 0.694 | Assumed to be included in the cost of CRS events |
| Neurological events grade 3/4 | 0.80 | 0.090 |  | 0.00 | 0.786 | Assumed to be included in the cost of CRS events |
| Diarrhoea | 0.103 | 0.010 | 0.029 | 1,814.41 | 0.286 | (29) |
| Dyspnoea | 0.115 |  | 0.011 | 1,799.56 | 1.286 | (27) |
| Febrile neutropenia | 0.39 | 0.100 | 0.014 | 5,878.85 | 1.031 | (30) |
| Gamma-glutamyl transferase increased | 0.07 |  |  | 329.99 | 2.514 | (28) |
| Hyperglycaemia | 0.047 |  |  | 392.98 | 0.286 | (28) |
| Hypertension | 0 |  | 0.008 | 2,944.48 | 0.771 | (29) |
| Hypokalaemia | 0.065 | 0.021 | 0.005 | 329.99 | 0.286 | (28) |
| Hyponatremia | 0.065 | 0.041 |  | 329.99 | 0.286 | Assumed the same as hypokalemia |
| Hypophosphatemia | 0.15 | 0.072 |  | 329.99 | 0.857 | Assumed the same as hypokalemia |
| ICANS | 0 | 0.165 |  | 7,748.90 | 0.571 |  |
| Leukopenia | 0.065 | 0.608 | 0.022 | 3,369.39 | 1.150 | (27) |
| Lymphopenia | 0.065 | 0.505 | 0.019 | 3,369.39 | 2.103 | (27) |
| Nausea | 0.10 | 0.010 |  | 1,765.53 | 1.286 | (27) |
| Neutropenia | 0.15 | 0.948 | 0.182 | 3,369.39 | 1.864 | (27) |
| Peripheral sensory neuropathy | 0.08 | 0.062 | 0.010 | 2,624.99 | 36.571 | (29) |
| Pneumonia | 0.19 | 0.103 | 0.053 | 9,654.83 | 0.929 | (31) |
| Pyrexia | 0.07 | 0.000 | 0.006 | 4,177.71 | 1.864 | (27) |
| Sepsis | 0.20 | 0.040 | 0.004 | 49,427.79 | 0.607 | (32) |
| Thrombocytopenia | 0.31 | 0.598 | 0.157 | 3,369.39 | 1.959 | (27)  9 |

Abbreviations: AIDPATH: Artificial Intelligence powered, Decentralized Production for Advanced Therapies in the Hospital; CAR-T: Chimeric antigen receptor T cells; SoC: standard of care; AE: adverse event; ST: Supraventricular Tachycardia; ICANS: Immune Effector Cell-Associated Neurotoxicity Syndrome.

1: The cost of AEs is determined by multiplying the cost of medication for each event by the rate at which the event occurs in patients and the timing of its occurrence (in model cycles). This process is applied consistently for AEs during CAR-T therapy, standard of care, and bridging chemotherapy. Since the occurrence rates of AEs for AIDPATH are not yet available, they are assumed to match those of Cilta-cel.

101=

Appendix E: Results of extrapolation, data fitting, and model selection for OS and PFS data

**Results of Model Selection**

The results of the AIC analysis, a mathematical method for evaluating how well a model fits the data it was generated from, along with visual inspections, show that the best-fitting parametric model for extrapolating OS and PFS data in this analysis is the log-normal distribution. See the Table S1 and Figure S1 and S2.

**Table S1**: AIC and BIC from the fitted parametric models to the data

| Distribution | AIC - OS | BIC - OS | AIC - PFS | BIC - PFS |
| --- | --- | --- | --- | --- |
| Exponential | 324.13 | 326.708 | 429.90 | 435.05 |
| Log-Normal | 324.56 | 329.712 | 431.23 | 438.96 |
| Log-Logistic | 324.82 | 329.967 | 431.67 | 434.24 |
| Gamma | 324.83 | 329.975 | 431.94 | 437.09 |
| Weibull (AFT) | 324.91 | 330.060 | 432.97 | 438.12 |
| Gompertz | 325.46 | 330.605 | 433.63 | 438.78 |
| Generalized Gamma | 326.51 | 334.232 | 433.67 | 438.82 |

Abbreviations: AIC: Akaike information criterion; BIC: Bayesian information criterion; OS: overall survival; PFS: progression-free survival

 **Figure S1**: Extrapolation overall survival

 

**Figure S2**: Extrapolation progression free survival

11=

**Mixture-cure**

Modelling the differences in survival between cured and uncured patients increased quality-adjusted life expectancy and costs in both arms as the long-term survival of cured patients was now accounted for.

After fitting the model of the uncured population to the Kaplan-Meier OS data, the results of the AIC analysis showed that the exponential mixture-cure distribution exhibited the lowest AIC, followed by the log-normal and gamma distributions (**Table S2**).  

**Table S2**: Model fitting to OS data

| Distribution OS | AIC fitted Parametric cure model | Cure fraction |
| --- | --- | --- |
| Exponential | 326.136 | 0.500 |
| Weibull | 326.854 | 0.508 |
| Log-Normal | 326.564 | 0.500 |
| Log-Logistic | 326.821 | 0.509 |
| Gompertz | 327.373 | 0.626 |
| Gamma | 326.777 | 0.565 |
| Generalized Gamma | 328.510 | 0.501 |

 Abbreviations: AIC: Akaike information criterion; OS: overall survival 

The fitting of a mixture-cure model to the PFS data showed the lognormal distribution to have the lowest AIC, followed by the log-logistic and the gamma distribution (**Table S3**).

12

Appendix F: Scenario analysis results

|  | Lower | Upper |
| --- | --- | --- |
| Discount rate outcomes | -2309.81 | -62927.6 |
| HR PFS standard of care vs CAR-T | -26549.1 | 8246.13 |
| Time horizon | -6421.31 | -3075.08 |
| Discount rate costs | 1392.88 | 4411.36 |
| Cytokine release syndrome grade 1/2 - Costs per AE | -3692.31 | -2325.69 |
| AIDPATH vein-to-vein time | -2435.11 | -3768.71 |
| Outpatient haematologist visit - Unit cost | -3610.4 | -2425.14 |
| HR OS standard of care vs CAR-T | -3860.22 | -2760.55 |
| Sepsis - Proportion of patients Cilta-cel | -3434.37 | -2502.73 |
| Peripheral sensory neuropathy - Proportion of patients SoC | -2834.55 | -3723.09 |
| Outpatient haematologist visit - Progression-free SoC | -3440.78 | -2631.08 |
| PFS utility (on-treatment) CARTITUDE-1 | -3427.89 | -2824.72 |
| Duration of CAR-T postmonitoring | -2770.86 | -3207.96 |
| Neutropenia - Costs per AE | -3257.29 | -2853.86 |
| Leukopenia - Costs per AE | -3249.26 | -2863.61 |

**Figure S3**: Most impactful parameters on the models output when comparing AIDPATH to SoC

131=

|  | Lower | Upper |
| --- | --- | --- |
| Discount rate outcomes | -1528017 | -34175759 |
| Comparator CAR-T vein-to-vein time | -7221142 | -1539742 |
| AIDPATH vein-to-vein time | -1311555 | -6671001 |
| Time horizon | -2940294 | -1986527 |
| Progressed disease utility | -2143118 | -1858945 |
| PFS utility (on-treatment) CARTITUDE-1 | -2119385 | -1884779 |
| Pre-infusion period utility | -1962199 | -2008600 |
| Outpatient haematologist visit - Unit cost | -1987301 | -1985586 |
| Outpatient haematologist visit - Progression-free SoC | -1986941 | -1986023 |
| Outpatient haematologist visit - Progressed (both) | -1986807 | -1986187 |
| Duration of CAR-T postmonitoring | -1986220 | -1986660 |
| Discount rate costs | -1989156 | -1989471 |
| Urinary light chain excretion - Unit cost | -1986663 | -1986361 |
| Urinary light chain excretion - Progression-free SoC | -1986663 | -1986361 |
| End of life costs | -1986405 | -1986674 |

**Figure S4**: Most impactful parameters on the models output when comparing AIDPATH to Cilta-Cel

141=

**Table S8**: Most impactful parameters on the models output when comparing AIDPATH to SoC

| Rank | Parameter | Lower ICER | Upper ICER | Difference |
| --- | --- | --- | --- | --- |
| 1 | Discount rate outcomes | -€2,310 | -€62,928 | €60,618 |
| 2 | HR PFS standard of care vs CAR-T | -€26,549 | €8,246 | €34,795 |
| 3 | Time horizon | -€6,421 | -€3,075 | €3,346 |
| 4 | Discount rate costs | €1,393 | €4,411 | €3,018 |
| 5 | Cytokine release syndrome grade 1/2 - Costs per AE | -€3,692 | -€2,326 | €1,367 |
| 6 | AIDPATH vein-to-vein time | -€2,435 | -€3,769 | €1,334 |
| 7 | Outpatient haematologist visit - Unit cost | -€3,610 | -€2,425 | €1,185 |
| 8 | HR OS standard of care vs CAR-T | -€3,860 | -€2,761 | €1,100 |
| 9 | Sepsis - Proportion of patients Cilta-cel | -€3,434 | -€2,503 | €932 |
| 10 | Peripheral sensory neuropathy - Proportion of patients SoC | -€2,835 | -€3,723 | €889 |

**Table S9**: Most impactful parameters on the models output when comparing AIDPATH to Cilta-Cel

151=

| Rank | Parameter | Lower ICER | Upper ICER | Difference |
| --- | --- | --- | --- | --- |
| 1 | Discount rate outcomes | -€1,528,017 | -€34,175,759 | €32,647,742 |
| 2 | Comparator CAR-T vein-to-vein time | -€7,221,142 | -€1,539,742 | €5,681,400 |
| 3 | AIDPATH vein-to-vein time | -€1,311,555 | -€6,671,001 | €5,359,446 |
| 4 | Time horizon | -€2,940,294 | -€1,986,527 | €953,768 |
| 5 | Progressed disease utility | -€2,143,118 | -€1,858,945 | €284,173 |
| 6 | PFS utility (on-treatment) CARTITUDE-1 | -€2,119,385 | -€1,884,779 | €234,606 |
| 7 | Pre-infusion period utility | -€1,962,199 | -€2,008,600 | €46,401 |
| 8 | Outpatient haematologist visit - Unit cost | -€1,987,301 | -€1,985,586 | €1,715 |
| 9 | Outpatient haematologist visit - Progression-free SoC | -€1,986,941 | -€1,986,023 | €918 |
| 10 | Outpatient haematologist visit - Progressed (both) | -€1,986,807 | -€1,986,187 | €620 |

References

1.	Latimer NR. NICE Decision Support Unit Technical Support Documents. Survival Analysis For Economic Evaluations Alongside Clinical Trials - Extrapolation with Patient-Level Data. London: National Institute for Health and Care Excellence (NICE)

Copyright © 2013 National Institute for Health and Clinical Excellence, unless otherwise stated. All rights reserved.; 2013.

2.	Rutherford MJ, Dickman PW, Coviello E, Lambert PC. Estimation of age-standardized net survival, even when age-specific data are sparse. Cancer epidemiology. 2020;67:101745.

3.	Jackson C, Thom H, Claeskens G. Different Models for Different Purposes: Focused Model Comparison in R.

4.	Ran T, Eichmüller SB, Schmidt P, Schlander M. Cost of decentralized CAR T-cell production in an academic nonprofit setting. International journal of cancer. 2020;147(12):3438-45.

5.	Jagannath S, Joseph N, Crivera C, Kharat A, Jackson CC, Valluri S, et al. Component Costs of CAR-T Therapy in Addition to Treatment Acquisition Costs in Patients with Multiple Myeloma. Oncology and therapy. 2023;11(2):263-75.

6.	Berdeja JG, Madduri D, Usmani SZ, Jakubowiak A, Agha M, Cohen AD, et al. Ciltacabtagene autoleucel, a B-cell maturation antigen-directed chimeric antigen receptor T-cell therapy in patients with relapsed or refractory multiple myeloma (CARTITUDE-1): a phase 1b/2 open-label study. Lancet (London, England). 2021;398(10297):314-24.

7.	GmbH NP. Tisagenlecleucel (Kymriah®) [German]. 2022.

8.	MP A. Medicinal Products Act in the version published on 12 December 2005 (Federal Law Gazette [BGBl.]) Part I p. 3394, last amended by Article 11 of the Act of 6 May 2019 (Federal Law Gazette I p. 646).

9.	Hecken. to the Resolution of the Federal Joint Committee (G-BA) on an Amendment of the Pharmaceuticals Directive (AM-RL): Annex XII – Benefit Assessment of Medicinal Products with New Active Ingredients According to Section 35a SGB V Polatuzumab Vedotin (Diffuse Large B-Cell Lymphoma, Combination with Bendamustine and Rituximab) 2020. Available from: <https://www.g-ba.de/downloads/40-1465-6796/2020-08-20_AM-RL-XII_Polatuzumab-Vedotin_D-507_TrG_EN.pdf>.

10.	Mitte KW. DRG-Entgelttarif 2022 für die Klinikum Würzburg Mitte gGmbH Stand 01.08.2022 [German]. Würzburg, Germany: KWM (Missioklinik / Juliusspital); 2022.

11.	Hecken. to the Resolution of the Federal Joint Committee (G-BA) on an Amendment of the Pharmaceuticals Directive (AM-RL): Annex XII – Benefit Assessment of Medicinal Products with New Active Ingredients According to Section 35a SGB V Daratumumab (New Therapeutic Indication: Multiple Myeloma, Newly Diagnosed, Patients Eligible for Autologous Stem Cell Transplant, Combination with Bortezomib, Thalidomide, and Dexamethasone)2020. Available from: <https://www.g-ba.de/downloads/40-1465-6794/2020-08-20_AM-RL-XII_Daratumumab_D-522_TrG_EN.pdf>.

12.	GmbH J-C. Daratumumab (Darzalex) {German]. 2018.

13.	Palumbo A, Bringhen S, Larocca A, Rossi D, Di Raimondo F, Magarotto V, et al. Bortezomib-melphalan-prednisone-thalidomide followed by maintenance with bortezomib-thalidomide compared with bortezomib-melphalan-prednisone for initial treatment of multiple myeloma: updated follow-up and improved survival. Journal of clinical oncology : official journal of the American Society of Clinical Oncology. 2014;32(7):634-40.

14.	Durie BGM, Hoering A, Abidi MH, Rajkumar SV, Epstein J, Kahanic SP, et al. Bortezomib with lenalidomide and dexamethasone versus lenalidomide and dexamethasone alone in patients with newly diagnosed myeloma without intent for immediate autologous stem-cell transplant (SWOG S0777): a randomised, open-label, phase 3 trial. Lancet (London, England). 2017;389(10068):519-27.

161=

15.	Usmani SZ, Weiss BM, Plesner T, Bahlis NJ, Belch A, Lonial S, et al. Clinical efficacy of daratumumab monotherapy in patients with heavily pretreated relapsed or refractory multiple myeloma. Blood. 2016;128(1):37-44.

16.	Chang HJ, Lee JH, Do YR, Bae SH, Lee JL, Nam SH, et al. A combination of melphalan, prednisone, and 50 mg thalidomide treatment in non-transplant-candidate patients with newly diagnosed multiple myeloma. The Korean journal of internal medicine. 2011;26(4):403-9.

17.	Moreau P, Masszi T, Grzasko N, Bahlis NJ, Hansson M, Pour L, et al. Oral Ixazomib, Lenalidomide, and Dexamethasone for Multiple Myeloma. The New England journal of medicine. 2016;374(17):1621-34.

18.	Broijl A, et al. Multipel Myeloom [Dutch] 2024 [25-09-2025]. Available from: <https://publicatie.hematologienederland.nl/richtlijnen/multipel-myeloom/>.

19.	Mateos MV, Masszi T, Grzasko N, Hansson M, Sandhu I, Pour L, et al. Impact of prior therapy on the efficacy and safety of oral ixazomib-lenalidomide-dexamethasone vs. placebo-lenalidomide-dexamethasone in patients with relapsed/refractory multiple myeloma in TOURMALINE-MM1. Haematologica. 2017;102(10):1767-75.

20.	Latif A, Kapoor V, Lateef N, Ahsan MJ, Usman RM, Malik SU, et al. Incidence and Management of Carfilzomib-induced Cardiovascular Toxicity; A Systematic Review and Meta-analysis. Cardiovascular & hematological disorders drug targets. 2021;21(1):30-45.

21.	Stewart AK, Rajkumar SV, Dimopoulos MA, Masszi T, Špička I, Oriol A, et al. Carfilzomib, lenalidomide, and dexamethasone for relapsed multiple myeloma. The New England journal of medicine. 2015;372(2):142-52.

22.	Dimopoulos MA, Lonial S, White D, Moreau P, Weisel K, San-Miguel J, et al. Elotuzumab, lenalidomide, and dexamethasone in RRMM: final overall survival results from the phase 3 randomized ELOQUENT-2 study. Blood cancer journal. 2020;10(9):91.

23.	San-Miguel JF, Hungria VTM, Yoon SS, Beksac M, Dimopoulos MA, Elghandour A, et al. Panobinostat plus bortezomib and dexamethasone: impact of dose intensity and administration frequency on safety in the PANORAMA 1 trial. British journal of haematology. 2017;179(1):66-74.

24.	Richardson PG, Attal M, Campana F, Le-Guennec S, Hui AM, Risse ML, et al. Isatuximab plus pomalidomide/dexamethasone versus pomalidomide/dexamethasone in relapsed/refractory multiple myeloma: ICARIA Phase III study design. Future oncology (London, England). 2018;14(11):1035-47.

25.	Palumbo A, Chanan-Khan A, Weisel K, Nooka AK, Masszi T, Beksac M, et al. Daratumumab, Bortezomib, and Dexamethasone for Multiple Myeloma. The New England journal of medicine. 2016;375(8):754-66.

26.	Kim SJ, Bang SM, Choi YS, Jo DY, Kim JS, Lee H, et al. Bendamustine in heavily pre-treated multiple myeloma patients: Results of a retrospective analysis from the Korean Multiple Myeloma Working Party. Blood research. 2016;51(3):193-9.

27.	Mickisch G, Gore M, Escudier B, Procopio G, Walzer S, Nuijten M. Costs of managing adverse events in the treatment of first-line metastatic renal cell carcinoma: bevacizumab in combination with interferon-alpha2a compared with sunitinib. British journal of cancer. 2010;102(1):80-6.

28.	Campone M, Yang H, Faust E, Kageleiry A, Signorovitch JE, Zhang J, et al. Cost of adverse events during treatment with everolimus plus exemestane or single-agent chemotherapy in patients with advanced breast cancer in Western Europe. Journal of medical economics. 2014;17(12):837-45.

29.	Wehler E, Zhao Z, Pinar Bilir S, Munakata J, Barber B. Economic burden of toxicities associated with treating metastatic melanoma in eight countries. The European journal of health economics : HEPAC : health economics in prevention and care. 2017;18(1):49-58.

30.	Ihbe-Heffinger A, Paessens B, Jacobs VR, Ehlken B, Shlaen M, Berger K, et al. Resource Use and Costs Associated with Routine Management of Febrile Neutropenia in German Hospitals. Blood. 2007;110(11):3338-.

171=

31.	Deb A, Podmore B, Barnett R, Beier D, Galetzka W, Qizilbash N, et al. Clinical and economic burden of pneumococcal disease among individuals aged 16 years and older in Germany. Epidemiology and infection. 2022;150:e204.

32.	Fleischmann C, Hartmann M, Hartog CS, Welte T, Heublein S, Thomas-Rueddel D, et al. Epidemiology of Sepsis in Germany: Incidence, Mortality And Associated Costs of Care 2007-2013: Intensive Care Med Exp. 2015 Oct 1;3(Suppl 1):A50. doi: 10.1186/2197-425X-3-S1-A50. eCollection 2015 Dec.

181=
